# Combating HIV stigma in low‐ and middle‐income healthcare settings: a scoping review

**DOI:** 10.1002/jia2.25553

**Published:** 2020-08-26

**Authors:** M. Kumi Smith, Richie H. Xu, Shanda L. Hunt, Chongyi Wei, Joseph D. Tucker, Weiming Tang, Danyang Luo, Hao Xue, Cheng Wang, Ligang Yang, Bin Yang, Li Li, Benny L. Joyner, Sean Y. Sylvia

**Affiliations:** ^1^ Division of Epidemiology & Community Health University of Minnesota Twin Cities Minneapolis MN USA; ^2^ Health Sciences Libraries University of Minnesota Twin Cities Minneapolis MN USA; ^3^ Department of Health Behavior, Society and Policy Rutgers University New Brunswick NY USA; ^4^ Institute for Global Health and Infectious Diseases School of Medicine University of North Carolina Chapel Hill NC USA; ^5^ London School of Hygiene and Tropical Medicine London UK; ^6^ The Zhitong LGBT Center Guangzhou China; ^7^ Freeman Spogli Institute for International Studies Stanford University Stanford CA USA; ^8^ Dermatology Hospital of Southern Medical University Guangzhou China; ^9^ Department of Epidemiology University of California Los Angeles CA USA; ^10^ Division of Pediatric Critical Care Medicine School of Medicine University of North Carolina Chapel Hill NC USA; ^11^ Department of Health Policy & Management University of North Carolina Chapel Hill NC USA

**Keywords:** HIV prevention, stigma, health systems, intervention, LMIC, public health

## Abstract

**Introduction:**

Nearly 40 years into the HIV epidemic, the persistence of HIV stigma is a matter of grave urgency. Discrimination (i.e. enacted stigma) in healthcare settings is particularly problematic as it deprives people of critical healthcare services while also discouraging preventive care seeking by confirming fears of anticipated stigma. We review existing research on the effectiveness of stigma interventions in healthcare settings of low‐ and middle‐income countries (LMIC), where stigma control efforts are often further complicated by heavy HIV burdens, less developed healthcare systems, and the layering of HIV stigma with discrimination towards other marginalized identities. This review describes progress in this field to date and identifies research gaps to guide future directions for research.

**Methods:**

We conducted a scoping review of HIV reduction interventions in LMIC healthcare settings using Embase, Ovid MEDLINE, PsycINFO and Scopus (through March 5, 2020). Information regarding study design, stigma measurement techniques, intervention features and study findings were extracted. We also assessed methodological rigor using the Joanna Briggs Institute checklist for systematic reviews.

**Results and discussion:**

Our search identified 8766 studies, of which 19 were included in the final analysis. All but one study reported reductions in stigma following the intervention. The studies demonstrated broad regional distribution across LMIC and many employed designs that made use of a control condition. However, these strengths masked key shortcomings including a dearth of research from the lowest income category of LMIC and a lack of interventions to address institutional or structural determinants of stigma. Lastly, despite the fact that most stigma measures were based on existing instruments, only three studies described steps taken to validate or adapt the stigma measures to local settings.

**Conclusions:**

Combating healthcare stigma in LMIC demands interventions that can simultaneously address resource constraints, high HIV burden and more severe stigma. Our findings suggest that this will require more objective, reliable and culturally adaptable stigma measures to facilitate meaningful programme evaluation and comparison across studies. All but one study concluded that their interventions were effective in reducing healthcare stigma. Though encouraging, the fact that most studies measured impact using self‐reported measures suggests that social desirability may bias results upwards. Homogeneity of study results also hindered our ability to draw substantive conclusions about potential best practices to guide the design of future stigma reduction programmes.

## 1 INTRODUCTION

Despite tremendous biomedical advances to make HIV more preventable [1, 2, 3] and more treatable [4], stigma is still cited as a significant barrier to controlling the epidemic. Stigma is defined as the social devaluation of a person based on an attribute [5], and discrimination is often described as the end result of stigmatization [6]. HIV stigma that unfolds in healthcare settings can be particularly harmful as it directly impacts public health outcomes. For example stigma by providers can result in service refusal, failure to offer HIV services, or negative clinical experiences that discourage future care seeking for those in need of services [7, 8, 9]. Among people living with HIV (PLWH), experiences of healthcare stigma have been associated with more adverse mental health outcomes [10, 11], poorer healthcare access [12, 13, 14] and more rapid disease progression [15, 16]. PLWH experiences of enacted healthcare stigma are widely documented across the globe.[17, 18, 19] Large‐scale stigma reduction efforts will need to be a cornerstone of planned global efforts to end the AIDS epidemic by 2030 [20].

Researchers have traditionally divided stigma into internalized, enacted, anticipated and perceived stigma. Internalized stigma or “self‐stigma” occurs when targets of stigma internalize the negative attitudes and perceptions projected onto them [21]. Enacted stigma refers to overt acts of discrimination and hostility directed at those perceived to have the stigmatized status, and anticipated stigma result from fear of enacted stigma [22]. Lastly perceived stigma pertains to how PLWH perceive their partners, friends, family and community to treat and view HIV and PLWH in general [6]. The various forms of stigma are often cyclical [23]: for example PLWH facing enacted stigma may be denied critical health services which may in turn validate their fears of anticipated stigma or expose them to negative experiences that deepen internalized stigma [21]. Interrupting stigma will therefore require interventions at multiple points in the cycle. Its inherently social nature will also require multi‐level interventions that target not only individual‐level behaviours but also change at the interpersonal, social, organizational and societal levels [24].

A rich history of HIV stigma interventions has been documented in four known reviews to date [25, 26, 27, 28]. Most employ a narrative approach to highlight the positive intervention effects reported by the majority of included studies, while also pointing out that these gains mask shortcomings in terms of short duration of effects [26], lack of methodological rigor [25] and inconsistent measures used across studies [28]. The fourth review, the only meta‐analysis, quantifies a significant but small effect across included studies to arrive at a similar conclusion regarding issues with study quality [27]. These four reviews are broadly inclusive of all studies on HIV stigma interventions regardless of the target population, intervention setting and global region. But their analyses do not focus on healthcare settings, the individuals who work in these settings, or on high priority regions.

This scoping review aims to expand on past reviews by assessing the design and impact of stigma reduction interventions conducted in healthcare settings of low‐ and middle‐income countries (LMIC). It also examines the methodological quality of these studies with particular attention to how stigma was measured. We opted to conduct a scoping approach (as opposed to other approaches such as a systematic review) in order to characterize the nature of evidence for these stigma interventions [29]. The substantial heterogeneity in approaches to measuring stigma and the interventions for reducing it presented challenges in specifying a clearly defined question (e.g. “does this strategy effectively reduce this specific outcome?”) also made a scoping review the more practical choice [30]. We focus on healthcare stigma in LMIC because of the unique combination of factors that shape stigma in these settings such as heavier HIV burden [31, 32], less developed primary healthcare systems [33, 34], and the layering of HIV stigma with discrimination towards other marginalized identities such as sexual minorities [35], commercial sex workers [36] or people who inject drugs [37]. In such settings, pursuit of global “best practices” for combating stigma can miss opportunities inadvertently results in the direct importation of western‐style interventions. By investigating studies in LMIC where the inherent challenges of stigma reduction are further compounded by resource constraints and other limitations, this review describes progress to date in the control of stigma in settings where these programmes may have the greatest impact.

## 2 METHODS

The Preferred Reporting Items for Systematic Reviews and Meta‐Analyses (PRISMA) Guidelines were followed to conduct the review and analysis [30].

### 2.1 Search strategy

A public health librarian (author SLH) created the literature search strategy after meeting with two members of the research team to clarify goals and further define selection criteria. The search strategy was built and tested for sensitivity in Embase using Emtree subject headings and keywords, and the search strategy was translated to three other databases: Ovid MEDLINE, PsycINFO and Scopus. There were no language or time restrictions placed on the search, which was conducted on 29 to 30 November 2018. Several additional texts were added in the course of revisions up through 5 March 2019.

Four conceptual domains were used to build the list of relevant search terms: health outcome (i.e. HIV), region (i.e. LMIC), key topic area (i.e. stigma in healthcare settings) and study design (i.e. intervention). Terms selected for each domain to include widely used language, acronyms and phrasings common in the medical and health sciences literature. Search terms defining the regional scope of the paper included a list of the 164 countries defined as low, low‐middle and upper‐middle income by the World Bank [38], as well as inclusion of a set of search terms commonly used to describe LMIC published by the Health Sciences Library at the University of North Carolina [39]. Terminology related to stigma and interventions were tested and reduced down to the terms in the Appendix S1 to reduce the sensitivity and increase the specificity of the search. Lastly, we screened the reference lists of selected articles to locate other potentially relevant studies and scanned the Internet for relevant grey literature including non‐peer‐reviewed reports or documents. Specific search terms used in the Embase search strategy are provided in the Appendix S1.

### 2.2 Study selection

Authors MKS and RHX reviewed the resulting titles and abstracts to identify articles that: (1) specified reduction in HIV‐related stigma in healthcare settings as at least one of the study aims, (2) included healthcare workers as members of the population targeted by the intervention and (3) took place in an LMIC as defined in the previous section. Note that while our topical interest centred on enacted stigma, we applied this criteria liberally in order to include studies that described issues of stigma in the context of patient‐provider encounters, regardless of whether the authors explicitly used the term “enacted stigma.” We defined healthcare workers as professionally trained medical personnel such as doctors, nurses, physician’s assistants, pharmacists, but not situationally trained lay workers such as community health workers or volunteer support staff. We drew this distinction because professional healthcare workers hold disproportionate power over their patients by way of their authority to prescribe (or withhold) life‐saving drugs, to perform (or abstain from) necessary medical procedures, or to refer patients to other medical specialists. Though practice around the inclusion of conference abstracts in scoping reviews vary, our team opted to include them in order to address the publication bias that tends to favour more frequent and more rapid publication of reports with positive results [40].

**Figure 1:**
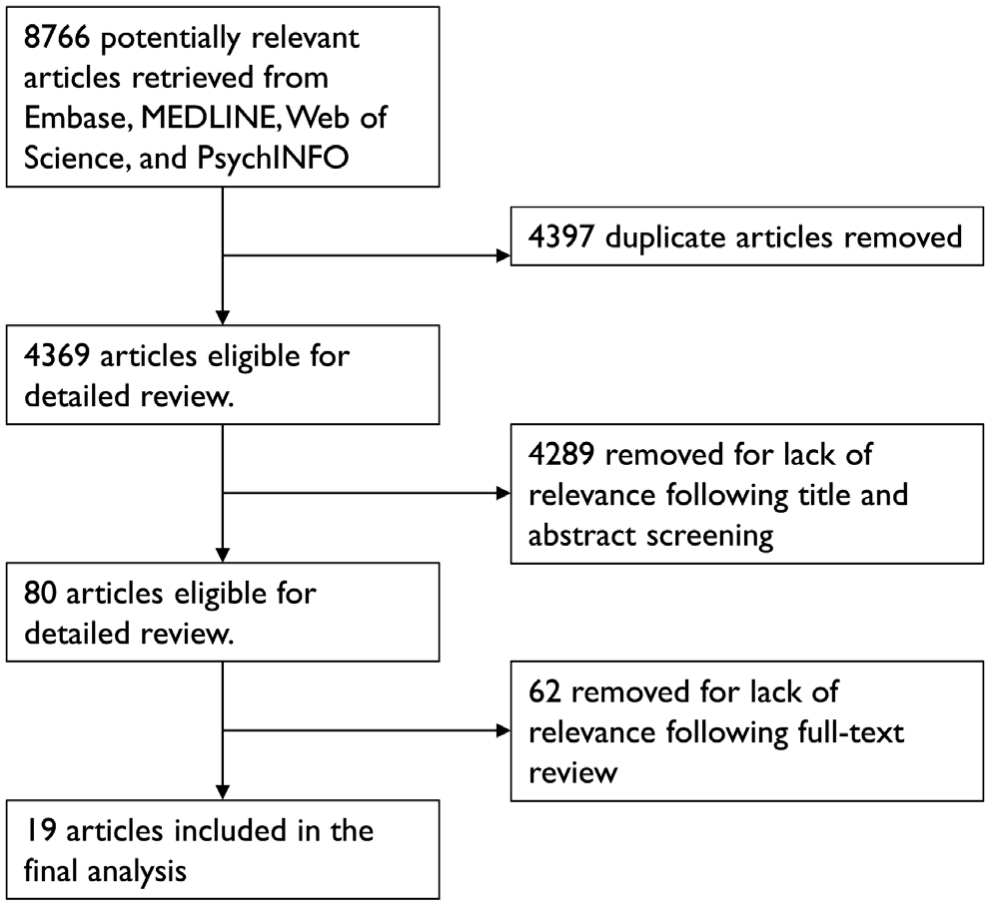
Flow diagram for study selection.

Next, the same two authors conducted a full‐text review to further determine its appropriateness for inclusion in the final analysis. In cases where multiple articles described the same intervention, only the reference with the most complete information was retained. All aspects of the review and text screening were carried out by authors MKS and RX independently in the software program Rayyan [41]. Discrepancies in decisions regarding article inclusion were resolved through discussion. Figure 1 shows the flow diagram for study selection.

### 2.3 Data extraction and bias assessment

Author MKS created an initial draft of a data extraction chart containing study features of interest a priori. Authors MKS and RHX then assessed chart utility by conducting data extraction for the first five articles, after which they compared results and amended the chart to better reflect study features and characteristics relevant to the analysis. A full data extraction was then carried out independently on each article by MKS and RHX, who met for a final time to resolve any differences in classifications.

The following data were extracted: study region, specific target population, sample size, study design, type of stigma measure, intervention characteristics and primary results. Approaches for stigma measurement were categorized into the three domains as identified by Nyblade *et al*. [42] and included providers’ awareness about the negative impacts of stigma, their knowledge about HIV transmission as it relates to fear of occupational exposure, moral or value‐based associations they have of HIV with socially taboo behaviours, and enacted stigma (i.e. actual measures of discriminatory behaviours on the part of providers).

Interventions were then categorized in three ways. First we determined the levels at which changes were sought: individual‐level strategies such as improving provider awareness of stigma versus institutional‐level changes such as implementation of guidelines for universal precautions. Second, we classified interventions using the four intervention types defined by Brown *et al*. [26]: information‐based training, skills building, contact with affected groups such as PLWH, and institutional changes such as altering clinic policy around PLWH care (e.g. ending the practice of segregating inpatients by HIV status). Last, we conducted a risk of bias assessment for all studies in the final sample set using the Joanna Briggs Institute checklist for systematic reviews [43]. Checklists specific to randomized control trials (RCT) and quasi‐experimental studies were applied separately to studies of corresponding design. Responses to each criteria were assessed using “yes” if criteria were met, “no” if they were not met, “N/A” if the question did not apply to the particular study, and “unclear” if the information could not be determined from the available text. We then assigned a final “overall rating” to each study based on the following algorithm: studies failing to meet more than two criteria were classified as “poor,” those that failed to meet exactly two criteria as “fair,” and less than two as “good.” Criteria classified as “N/A” or “unclear” were treated as equivalent to half of a “no;” for example a study whose criteria rating included a single “no” and two “unclears” would receive an overall rating of “fair.”

## 3 RESULTS AND DISCUSSION

The initial search produced 8766 results (Embase = 2605; Ovid Medline = 2215; PsycINFO = 1089; Scopus = 2857), and after removing duplicates, 4369 studies remained. Review of the titles and abstracts identified 80 studies for full‐text examination, after which 19 studies were found to meet criteria regarding the outcome of interest (enacted healthcare stigma), the study population (healthcare workers) and study design (intervention; Figure 1). Most of the studies were published as full articles in peer reviewed journals [44, 45, 46, 47, 48, 49, 50, 51, 52, 53, 54, 55, 56, 57], with the remainder published as conference abstracts [58, 59, 60] or public reports [61, 62, 63] (Table 1).

**Table 1:** Characteristics of included studies

Authors (year)	Country/cities	Target population	Sample size	Study design	Stigma measurement	Intervention types, levels, development	Primary results
Geibel *et al*. (2017) [44]	Bangladesh	Physicians, nurses, paramedics, counsellors	300 Individuals	Single group pre/post‐test design	24‐item scale to measure stigma in values/judgement domain. Scale adapted from one previously used in two other non‐Western settings [64, 65]; no information about scale validation provided. Clinic attendees were also surveyed after the intervention to assess their satisfaction with clinic services	2 (IB, SB); individual and institutional‐level; intervention based on guide developed by the International HIV/AIDS Alliance [66]	Among providers, lower likelihood post‐intervention to endorse 3 of the 24 stigma scale items; patient clients reported less enacted stigma post‐intervention
Diesel *et al*. (2013) [45]	Cameroon	Nursing students	41 Individuals	Single group pre/post‐test design	Five scales used to measure stigma in the domains of domains of stigma awareness, HIV knowledge and values/judgement. Instruments included: (1) HIV/AIDS Stigma Instrument [47]; (2) Nurse Willingness Questionnaire [67]; (3) AIDS Attitude Scale [68] (4) AIDS Knowledge Scale [69]; and the (5) Obstetrical Knowledge Scale (developed by the authors). Content validity and reliability statistics of all but the final scale cited from the original studies	2 (IB, SB); individual‐level; intervention content informed by formative research by authors	Provider knowledge, attitudes and action plans improved but mixed results regarding perception of stigma
Li *et al*. (2014) [50]	China	Physicians	40 Clusters	Cluster‐RCT	Standardized patients conducted unannounced clinic visits as PLWH to rate providers on general service satisfaction, perceived attitudes and compliance with universal precautions such as glove wearing or proper disposal of needles [70, 71]. Domain of enacted stigma; no details on development of client satisfaction score provided	2 (IB, SB); individual and institutional –level; intervention developed based on diffusion of innovation theory [72]	Providers in intervention hospitals received higher satisfaction ratings than those in control hospitals
Wang *et al*. (2009) [51]	China	Physicians and patients in primary care clinics	69 Individuals	Single group pre/post‐test design	Assessments to measure physician knowledge regarding HIV biology and HIV‐related stigma (domains: stigma awareness; HIV knowledge), presumably developed by authors. Patients also surveyed on knowledge of HIV and stigma. No details on assessment tools provided	2 (IB, SB); individual‐level; intervention developed by US/China medical team in iterative process over the course of a year	Physician knowledge improved, including knowledge on stigma and discrimination. Among patients, HIV knowledge, stigmatizing attitudes and HIV testing improved after the intervention
Williams *et al*. (2006) [52]	China	Nurses	208 Individuals; 7 clusters	Single group pre/post‐test design	Three scales to measure stigma in domains of HIV knowledge and stigma awareness. The AIDS Attitude Scale to measure attitudes towards PLWH [68] (24 items) [73], avoidance and empathy (21 items) [74] and willingness to treat (13 items) [67]. Content validity and reliability statistics cited from original study; only one of three scales modified for use in local setting	2 (IB, SB); individual‐level; details on intervention development not provided	
Wu *et al*. (2008) [75]	China	Physicians, nurses, laboratory technicians	138 Individuals	RCT	Three‐item scale to measure stigma in domains of stigma awareness; no details of development or validity/reliability provided. Single question for domain of HIV knowledge adapted from US‐based workshop in 2004; authors note superior validity of the question from original study [70, 75]	3 (IB, SB, CWAG); individual‐level; intervention design modified using input from local community advisory board	Greater reduction in stigmatizing attitudes among providers in the intervention versus control arm. Not all reductions were sustained past the 3‐month follow‐up
Lohiniva *et al*. (2016) [53]	Egypt	Physicians, nurses	347 individuals; 2 clusters	Cluster‐control group pre/post‐test design	27‐item scale to measure stigma in domains of HIV knowledge and values/judgement. Adapted from two scales developed for non‐Western settings [76, 77] and modified for study setting in consultation with local providers	3 (IB, SB, CWAG); individual‐level; intervention developed using participatory approach with local task force	Greater reductions in both value‐based and fear‐based stigma scores in intervention versus control hospital. Reductions were particularly pronounced among nurses
Nyblade (2018) [63]	Ghana	“Facility staff and manage‐ment.”	Two separate surveys of 1149 (pre‐test) and 1149 (post‐test); individuals; 10 clusters	Cluster‐control group pre/post‐test design	Six‐item assessment tool to measure stigma in domains of HIV knowledge and values/judgement, presumably developed by authors. No other details on assessment tools provided	2 (IB, SB); individual and institutional‐level; intervention development informed by baseline data and in collaboration with local partners	Reductions in measures of HIV‐related fear and stigmatizing attitudes, though reductions lacked statistical significance for 2 of the 6 items
Allam *et al*. (2016) [60]	India	“Healthcare providers.”	Unknown number of individuals; 20 clusters; 117 post‐project interviews	Single group pre/post‐test design	An “adapted questionnaire” and qualitative methods used to obtain stigma measures in domains of HIV knowledge. Follow‐up surveys with clients to assess implementation of stigma‐free practices (domain: enacted stigma). No other details on assessment tools provided	2 (IB, institutional changes^a^); individual and institutional‐level; details on intervention development not provided	Qualitative data suggested less fear towards patients with HIV and strong follow through on institutional changes. Quantitative data collected from 117 in‐patients at a subset of the institutions suggested strong ongoing adherence to instituted changes
Mahendra *et al*. (2006) [62]	India	Physicians, nurses, “ward staff”	Two separate surveys of 884 (pre‐test) and 885 (post‐test) individual; 3 clusters	Single group pre/post‐test design	21‐item index to measure stigma in domains of HIV knowledge and values/judgement, Adapted from a “review of the national and international literature” and pre‐tested at the study site; qualitative interviews with hospital managers	4 (IB, SB, CWAG, institutional changes^a^); individual and institutional –level; intervention design informed by formative research and results of baseline survey	Statistically significant reduction in mean scores on the stigma index following the intervention
Pisal *et al*. (2007) [54]	India	Nurses.	371 Individuals	Single group pre/post‐test design	96 item questionnaire to assess stigma in domains of HIV knowledge, stigma awareness and values/judgement. Items were developed by authors and pre‐tested to ensure clarity of questions	3 (IB, SB, CWAG); individual‐level; Intervention content from the Population Council/Horizons and Sharan (India) were modified by a interdisciplinary team from the US and India	The overall percentage of high levels of fear of infection were reduced from 22% to 6%. In cases in which protective measures were not available, reduction in high levels of fear dropped from 74% to 50%
Shah *et al*. (2014) [55]	India	Nursing students.	91 individuals	Control group pre/post‐test design	29‐item scale to measure stigma in domains of HIV knowledge, stigma awareness and values/judgement. Developed previously for use in the US and India [78, 79, 80]; details on modification, validity, etc. not provided	2 (IB, CWAG); individual‐level; intervention curriculum adapted from resources of the International Center for Research on Women	Greater improvements in HIV‐related knowledge and reduction in stigma measure in the intervention versus control group. Changes in several fear‐related scale items lacked statistical significance
Edwards *et al*. (2016) [56]	Jamaica, Kenya, Uganda, South Africa	Nurses	813 Individuals; 90 clusters	Control group pre/post‐test design	Adaptation of the HIV‐AIDS Stigma Instrument–Nurse (HASI‐N) [81] to measure in domains of stigma awareness. Reduced from 33 to 19 items). Details on modification or scale validity, etc. not provided	2 (IB, SB); individual‐level; centralized model adapted to each of the 5 study settings guided by a local panel	Greater decreases in stigmatizing attitudes towards PLWH in intervention versus control arm, except in one setting where decreases were greater in the control arm. Only one site reported greater decreases in nurses experiencing stigma in the intervention versus control arm
Kaponda *et al*. (2009) [57]	Malawi	Clinical and non‐clinical hospital workers	855 individuals	Single group pre/post‐test design	Combination of a 6‐item scale (source of scale not described) and a single item from the Malawi Demographic Health Survey to measure stigma in HIV knowledge domain. Scale modified using psychometric analysis and respondent input	2 (IB, SB); individual‐level; some content based on formative work by the authors [82]	Decreases in one of the HIV‐related stigma items (PLWH are to blame for their infection) but no change in the other (PLWH should be permitted in public spaces or to cook for others)
Mbeba *et al*. (2011) [46]	Malawi	Clinical and non‐clinical hospital workers	336 Individuals; 2 clusters	Control group pre/post‐test design	Two scales to measure stigma in the domains of HIV knowledge and stigma awareness. Scales were adapted from authors’ previous work in same setting and from Demographic Health Survey; authors assessed internal consistency and correlation across measures	2 (IB, SB); individual‐level; structure based off previous HIV prevention in Africa [83, 84] and the WHO primary care model [85]	Greater reductions in stigmatizing attitudes towards PLWH in the intervention versus control arm, though the difference did not attain statistical significance until 30‐months post‐intervention (the final of three time points)
Uys *et al*. (2009) [47]	Malawi, South Africa, Swaziland, Tanzania, Lesotho	Nurses.	84 Individuals	Single group pre/post‐test design	HIV‐AIDS Stigma Instrument–Nurse (HASI‐N [81]; 19 items) and the HIV‐AIDS Stigma Instrument–PLWA (HASI‐P [86]; 33 items). Both scales measure stigma in domains of stigma awareness. Content validity and reliability statistics cited from original study	2 (IB, CWAG); individual‐level; teams of PLWH and nurses developed unique interventions in each country setting in two days	No change in the stigma scores between baseline and follow‐up, but a larger portion of participants were tested for HIV following the intervention
Ezedinachi *et al*. (2002) [48]	Nigeria	Physicians, nurses, laboratory technicians	1556 Individuals	RCT	Two indices to capture stigma in domains of values/judgement and HIV knowledge. Indicators developed by authors using findings from the formative phase of research; questions pilot tested for clarity	2 (IB, SB); individual‐level; details on intervention development not provided	Reductions in stigma scores following the intervention related to fear, compassion and willingness to care for PLWH. No change in many items related to clinical skills such as ability to counsel about drug use or safe sex
Nwuba *et al*. (2013) [59]	Nigeria	“Health workers.”	Unknown number of individuals; 4 clusters	Single group pre/post‐test design	“Health worker attitude” assessed using unknown methods; no domain or modification information provided. Measures presumably developed by the authors	4 (IB, SB, CWAG, institutional changes^a^); individual and institutional‐level; details on intervention development not provided	Method of analysis not provided, but authors conclude that “health worker attitude improved significantly”
Nanayakkara *et al*. (2016) [58]	Sri Lanka	Nursing students	129 Individuals	Control group pre/post‐test design	Two scales to measure stigma in domains of HIV knowledge and values/judgement, presumably developed by the authors. No other details on assessment tools provided	2 (IB, CWAG); individual‐level; details on intervention development not provided	Greater improvements in both knowledge and attitude scales in the intervention group. Changes in some items on the sub‐scales lack statistical significance
Pulerwitz *et al*. (2015) [49]	Vietnam	“Hospital staff”	797 Individuals; 4 clusters	Two‐armed pre/post‐test design	Three scales adapted from previous studies in other LMIC to measure stigma in the domains of HIV knowledge and values/judgement [77, 87]. Scale modification conducted in consultation with local programme staff and based on observation of local healthcare settings	2 (IB, SB); individual‐level; intervention developed based on teams’ past work in India [62] and from the ISDS stigma toolkit [88]	Significant reductions in stigmatizing attitudes and discriminatory behaviours in both arms following the intervention (Arm 1 addressed fear‐based stigma; Arm 2 address both fear‐based and social stigma)
Kiragu *et al*. (2008) [61]	Zambia	Physicians, nurses, clinical officers, paramedics, administrative staff, students	Two separate surveys of 1424 (pre‐test) and 1336 (post‐test) individual; 2 clusters	Control group pre/post‐test design	Six‐item scale to measure stigma in the domain of HIV knowledge and values/judgement, presumably developed by the authors. No details on scale development or sources	2 (IB, SB); individual‐level; details on intervention development not provided	Participation in the intervention was significantly associated with lower stigma scores, greater HIV knowledge and HIV testing

CWAG, contact with affected groups; IB, information‐based approaches; SB, skills‐based approaches.

^a^ Institutional changes included policies such as desegregation of HIV and non‐HIV patient hospital beds, new policies prohibiting the indication of HIV status on hospital records, or implementation of universal precautions for HIV.

**Figure 2:**
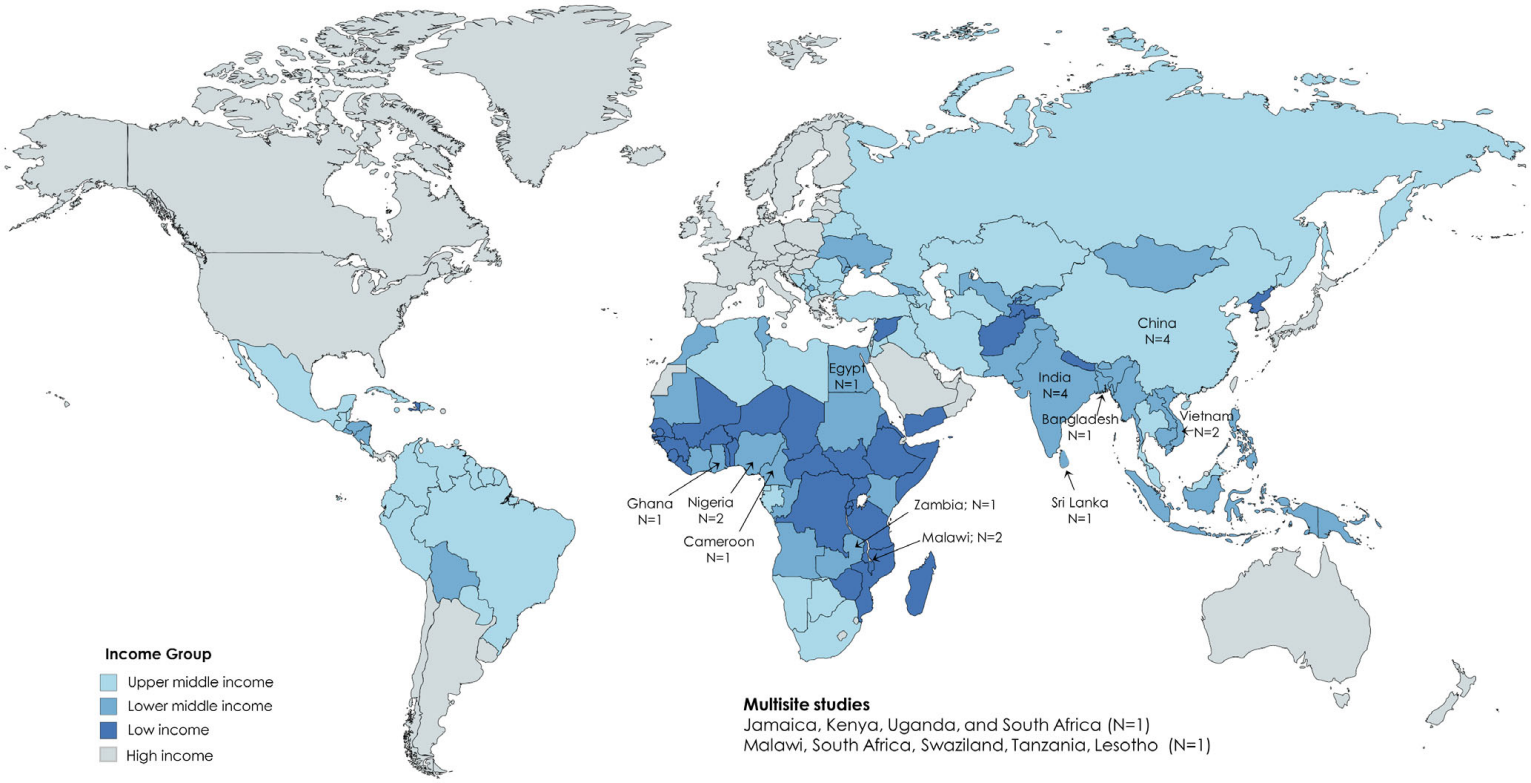
World map indicating locations from which studies included in this review were conducted. Colours indicate the World Bank income classifications. Note that studies from high‐income countries were not included in this review.

According to the World Bank geoscheme [89], a third of studies were located in sub‐Saharan Africa [45, 48, 57, 59, 61, 63, 83] followed by South Asia [44, 54, 55, 58, 60, 62]. Among the 10 studies located in East Asia and the Pacific, six took place in China and India which account for over half of people living with HIV in the region [50, 51, 52, 75]. Only one study was conducted in the Middle East and Northern Africa [53] (Figure 2). Additionally, two studies had sites in multiple countries, most of which were collectively located in sub‐Saharan Africa [47], but one of which also had a single site in Latin American and the Caribbean [56]. The most common study design was a single group pre/post‐test comparison [44, 45, 47, 51, 52, 54, 57, 59, 60, 62, 63] followed by pre/post‐test comparisons with a control group [46, 49, 55, 56, 58, 61, 63] (including one that used a comparative effectiveness trial design [49]) and RCTs [48, 50, 75].

### 3.1 Intervention strategies and impact

Interventions conducted by every study in this review focused on the individual‐level changes which included strategies such as improvement of HIV knowledge or reduction in discriminatory attitudes. Among these, six studies [44, 50, 59, 60, 62, 63] additionally included strategies focused on institutional‐level change, such as implementation of universal precautions, provision of preventive medical equipment like gloves or sharps containers and reversal of discriminatory clinical practices such as marking patient medical records to signal their HIV status to colleagues.

Using the classification strategy introduced by Brown *et al*., every study in this review was found to have employed at least two strategies as part of the intervention, with some using three [53, 54, 75] or at most four [59, 62] approaches in the same study. Information‐based training was included in every study, an approach most commonly deployed through didactic lectures or group discussion. Skills‐building was used in all but three of the studies [47, 55, 60] and involved techniques such as role playing, group brainstorming or sharing of personal experiences. Contact with affected groups was used in less than half [47, 53, 54, 55, 58, 59, 62, 75] of studies, and institutional changes such as desegregation of patients living with HIV from other patients or implementation of universal precautions was used in five studies [44, 50, 59, 60, 62]. Physicians and nurses made up the intervention target population of every study – including students still in training for medicine and nursing – among which half [44, 46, 48, 49, 51, 57, 59, 61, 62, 63, 75] also included nonclinical staff such as laboratory technicians or administrative staff in their intervention activities.

Regarding impact, all but one study [47] reported that their intervention was effective in reducing HIV stigma in healthcare settings. However, in 10 of the studies [44, 45, 48, 55, 56, 57, 58, 59, 63, 76], the overall conclusion was drawn from selectively focusing on the subset of stigma scale items where a significant effect was observed. In these cases, changes in the other scale items either lacked statistical significance or in one case the control condition experienced greater stigma reduction than the intervention [56].

### 3.2 Stigma measurement

As a function of our study inclusion criteria, all interventions in this review addressed stigma enacted by healthcare providers in healthcare settings. Yet measures varied widely in terms of measurement development, the groups surveyed and the domains assessed (i.e. stigma awareness, HIV knowledge, value‐based judgements and performance). A summary of measure characteristics is provided in Table 3.

In terms of stigma scale development, about half used scales or indices developed by the authors themselves in the course of the study [46, 48, 50, 51, 57, 58, 59, 61, 63]. Of these, three described steps taken to validate the measure, whether by conducting psychometric analyses of the scales [46, 57] or by pilot‐testing questions with local community members [48]. The remainder of studies relied on existing stigma measurement tools which were either used “as is” [44, 45, 47, 52, 55, 56, 60, 75] or with modifications informed by consultation with local experts [53, 62, 63] or by pre‐testing the instrument in target communities [49, 54].

The most common group surveyed was providers whose data were used to inform measures of stigma in all but two studies [50, 60]. In nearly all studies where providers were surveyed [44, 46, 48, 49, 51, 52, 53, 54, 55, 57, 58, 59, 61, 62, 63, 75], measures assessed providers’ responses to questions on factors such as their degree of comfort treating PLWH, their endorsement of coercive measures towards PLWH, beliefs regarding PLWH responsibility for their infection status, or other related items. The remaining three studies that surveyed providers approached them as potential witnesses to discriminatory behaviours taking place in their own workplace using the HIV/AIDS Stigma Instrument (HASI) [45, 47, 56]. The next most common group surveyed were patients who were asked either as clients evaluating the quality of care received at study clinics [44, 60], as witnesses to discriminatory treatment in clinics [47], or as future patients in term of their willingness to seek HIV‐related care in a study clinic following the intervention [51]. Each of these four studies utilized patient‐related measures in conjunction with those for providers. In two final studies, study investigators and staff directly observed provider behaviours to assess evidence of stigma reduction. Allam *et al*. [60] observed the persistence of recommended practices in study clinics such as removing HIV status from patient case records or integrating hospital wards for inpatients with and without HIV. Li *et al*. [50] used an unannounced standardized patient approach to observe and compare provider behaviours before and after the intervention.

Regarding stigma domains, the most commonly measured domain was HIV knowledge [45, 46, 48, 49, 51, 52, 53, 54, 55, 57, 58, 60, 61, 62, 63], followed by value‐based judgements [44, 45, 48, 49, 53, 54, 55, 58, 61, 62, 63], stigma awareness [45, 46, 47, 51, 52, 54, 55, 56, 63, 75] and enacted stigma [44, 45, 47, 49, 50, 51, 56, 60]. We were unable to ascertain the stigma measurement tool used in just one of the studies [59]. Most studies utilized measures that simultaneously captured multiple domains; at most three domains were measured in the same study [45, 54, 55].

### 3.3 Quality assessment

Appraisal of study quality was restricted to full reports (i.e. all but the three conference abstracts [58, 59, 60]) and conducted separately depending on whether it was an RCT or quasi‐experimental study. The most common weakness of the three RCTs included in this review [46, 48, 75] (Table 2A) was the lack of blinding of participants or study staff to treatment allocation, an expected result given the nature of behavioural interventions. Among the 15 quasi‐experimental studies (Table 2B) the most common criteria that studies failed to meet was inclusion of a control group [44, 45, 51, 52, 54, 57, 62] and completeness or the reporting of participant follow‐up over time [45, 51, 52, 53, 57, 60, 63]. Many of the quasi‐experimental studies also lacked adequate detail to inform appraisal by our reviewers (MKS and RHX), particularly in regards to reliability of the outcome measures. It is worth noting that the Li *et al*. [50] study using standardized patients, though classified here as a quasi‐experimental design, was conducted in the context of a larger RCT that evaluated the intervention using survey‐based stigma outcome measures [90] but was not included in this review to avoid redundancy.

**Table 2:** Risk of bias assessment for randomized control trials (A; N = 3) and quasi‐experimental studies (B; N = 16)

A. Randomized control trials			
Authors	Ezedinachi *et al*. (2002) [48]	Mbeba *et al*. (2011) [46]	Wu *et al*. (2008) [75]
Was true randomization used for assignment of participants to treatment groups?	Yes	No	Yes
Was allocation to treatment groups concealed?	No	No	No
Were treatment groups similar at the baseline?	Yes	Unclear	Yes
Were participants blind to treatment assignment?	No	No	No
Were those delivering treatment blind to treatment assignment?	No	No	No
Were outcomes assessors blind to treatment assignment?	No	No	No
Were treatment groups treated identically other than the intervention of interest?	Yes	Yes	No
Was follow‐up complete and if not, were differences between groups in terms of their follow‐up adequately described and analysed?	No	No	Yes
Were participants analysed in the groups to which they were randomized?	Yes	Yes	Yes
Were outcomes measured in the same way for treatment groups?	Yes	Yes	Yes
Were outcomes measured in a reliable way?	No	Yes	Yes
Was appropriate statistical analysis used?	Yes	Yes	Yes
Was the trial design appropriate, and any deviations from the standard RCT design (individual randomization, parallel groups) accounted for in the conduct and analysis of the trial?	Yes	Yes	Yes
Overall rating	Fair	Poor	Fair

B. Quasi‐experimental studies										
	Is it clear in the study what is the cause and what is the effect (i.e. there is no confusion about which variable comes first)?	Were the participants included in any comparisons similar?	Were the participants included in any comparisons receiving similar treatment/care, other than the exposure or intervention of interest?	Was there a control group?	Were there multiple measurements of the outcome both pre and post the intervention/ exposure?	Was follow‐up complete and if not, were differences between groups in terms of their follow‐up adequately described and analysed?	Were the outcomes of participants included in any comparisons measured in the same way?	Were outcomes measured in a reliable way?	Were appropriate statistical analysis used?	Overall rating
Geibel *et al*. (2017) [44]	Yes	N/A	N/A	No	Yes	Yes	Unclear	Yes	Yes	Poor
Diesel *et al*. (2013) [45]	Yes	N/A	N/A	No	No	No	N/A	Yes	Yes	Poor
Wang *et al*. (2009) [51]	Yes	N/A	N/A	No	No	No	N/A	Unclear	Yes	Poor
Li *et al*. (2014) [50]	Yes	Yes	Yes	Yes	No	Yes	Yes	Yes	Yes	Good
Williams *et al*. (2006) [52]	Yes	N/A	N/A	No	No	No	N/A	Yes	Yes	Poor
Lohiniva *et al*. (2016) [53]	Yes	Yes	Yes	Yes	No	No	Yes	Yes	Yes	Fair
Nyblade (2018) [63]	Yes	Yes	Yes	Yes	No	No	Yes	Yes	Yes	Good
Mahendra *et al*. (2006) [77]	Yes	N/A	N/A	No	No	Yes	N/A	Yes	Yes	Poor
Pisal *et al*. (2007) [54]	Yes	N/A	N/A	No	No	No	N/A	Yes	Yes	Poor
Shah *et al*. (2014) [55]	Yes	Yes	Yes	Yes	No	Yes	Yes	Yes	Yes	Good
Edwards *et al*. (2016) [56]	Yes	No	Yes	Yes	No	Yes	Yes	Yes	Yes	Good
Mbeba *et al*. (2011) [46]	Yes	Unclear	Yes	Yes	Yes	Yes	Yes	Unclear	No	Fair
Kaponda, *et al*. (2009) [57]	Yes	N/A	N/A	No	No	No	Unclear	Yes	Yes	Poor
Pulerwitz *et al*. (2015) [49]	Yes	Yes^a^	Yes	Yes^a^	Yes	Yes	Yes	Yes	Yes	Good
Kiragu (2008) [61]	Yes	Yes	Unclear	Yes	No	Yes	Yes	Yes	Yes	Fair

^a^ These studies were designed as two‐arm trials comparing two experimental conditions, which in this table was considered.

### 3.4 Summary of findings

This scoping review describes a body of rich body of research by adding 10 new studies to the collective body of research identified by earlier reviews on this topic [25, 26, 27, 28]. This body of research exhibits some key strengths. First, the 21 studies included in this review represent LMIC across five of the seven World Bank regions [38], nine of which were located in or had at least one site in sub‐Saharan Africa [45, 46, 47, 48, 56, 57, 59, 61, 63] which bears 70% of the global burden of HIV [91]. Regarding study design over half of the sample used a control condition [46, 49, 53, 55, 56, 58, 61, 63], including three RCTs [46, 48, 75]. Every intervention in this review employed at least two intervention components, and seven studies utilized stigma measures that had been developed or modified to account for the local cultural context. Lastly, all but one [47] of the 21 studies included in this review reported a significant effect of the intervention in reducing stigma in healthcare settings.

Though the consistency of positive results across these studies is encouraging, several features of these studies as a group merit discussion. First, we observed a dearth of studies taking place within the lowest income category of LMIC, as well as in Eastern Europe & Central Asia and Latin America & the Caribbean, regions where HIV incidence is rising [92, 93] or persistent at high levels [94, 95]. Second and in terms of interventions, relatively few (N = 6) sought to address institutional or structural determinants of stigma [44, 50, 59, 60, 62, 63]. And although the fact that many interventions combine multiple components can be seen as a strength, this can also hinder investigators’ ability to identify the most impactful activities, an insight which could inform stigma control efforts under budget constraints. Third, methodological quality across studies varied widely, with only five studies ranked as “good” [49, 50, 55, 56] and over half ranked as “poor” [44, 45, 46, 51, 52, 54, 58, 59, 60, 62, 96]. Lastly, the homogeneity of the findings that nearly all tested intervention were effective, while encouraging, may be a byproduct of the upward bias that can be induced with the use of self‐reported outcomes that trigger social desirability bias. The uniformity of results also hindered our ability to draw substantive conclusions about specific best practices to inform future stigma reduction programmes. Qualitative assessment of the relative magnitudes of effect may potentially inform a few such insights, though the one meta‐analysis on this topic conducted by Mak *et al*. cited a number of challenges to synthesizing findings including the “diversity of outcome measures used” [27].

The inherent challenges of stigma measurement are a topic of longstanding debate [22, 42, 77, 97] and merit special consideration. As a multifaceted phenomenon, stigma measurement demands complex tools to operationalize its myriad domains. Yet the tremendous diversity in the measures across the studies in this review – scale components range from three [75] to 96 [54] items – undermines our ability draw greater insight into the state of stigma reduction as a whole. In addition, measurement of some stigma domains – namely HIV knowledge and stigma awareness – commonly rely on use of hypothetical questions. This technique tests the underlying assumption that stigma is driven by ignorance of its effects on its targets or by fear of accidental transmission. But hypothetical questions are likely a poor proxy for how respondents behave in real life [97] and can lead to ambiguous interpretations, particularly in certain language groups and cultures [98]. Lastly, though enacted stigma is challenging to measure given low provider willingness to self‐report discriminatory behaviours and their tendency to alter behaviours under observation [42, 99], it can also provide the most substantive metric to assess whether stigma reduction interventions achieved their goals. Several measures of enacted stigma identified in this review sidestepped the problem of social desirability bias by using techniques other than self‐reported measures, whether by asking providers [45, 47, 56] and/or patients [44, 45, 47, 60] to bear witness to enacted stigma, or by deploying standardized patients to covertly observe provider behaviours [50]. Though these measures are not without their own limitations – for example the HASI‐N may inadvertently solicit multiple reports of the same stigma incident – their novelty and potential for reducing social desirability bias may motivate more research to further refine and improve techniques like these. A summary of these techniques and their potential limitations are provided in Table 3.

**Table 3:** Summary of non‐self‐reported measures of enacted stigma used among a subset of studies included in this scoping review

Measure	Description	Potential weaknesses
Client satisfaction survey [44, 60]	Patients are surveyed before and after an intervention to assess their experience with providers and satisfaction with care	Client satisfaction can be affected by many factors beyond provider control such as a patient’s baseline health, their treatment outcomes, or costs of care
HIV/AIDS Stigma Instrument‐PLWA (HASI‐P) [47]	A validated instrument administered to PLWH asking them to report on personal experiences of stigma such as verbal abuse or health care neglect	Patient perceptions of medical maltreatment or neglect may be shaped by asymmetric information regarding the appropriate course of treatment for their given condition
HIV/AIDS Stigma Instrument‐Nurse (HASI‐N) [45, 47, 56]	A validated instrument administered to healthcare providers asking them to report on instances when colleagues have stigmatized HIV patients or when they were stigmatized against for working with HIV patients	Possibility that multiple participants may report on the same instance of enacted stigma, which could lead to over‐estimation of stigma in a particular healthcare setting
Standardized patients [50]	Trained standardized patients make unannounced clinic visits to observe behaviours and clinical performance	The need to obtain informed consent from providers prior to visits could increase risk of less stigmatizing doctors selecting into the study (selection bias)

## 4 CONCLUSIONS

Our findings highlight several important knowledge gaps to guide future research directions. First, more research is needed to push the development of objective, reliable and transportable stigma measures to facilitate more meaningful programme evaluation and comparison across studies. Second, a common design weakness of studies in this review is the inability to blind participants or study staff to the intervention during the trial (see Table 2A). The modified Zelen design [100, 101] may offer a partial solution to this problem by not informing control arm participants of their trial participation until the study end. Finally, though many incorporated studies address attitudes towards key populations or HIV‐related risk behaviours (e.g. injection drug use, commercial sex, same sex behaviours), they do not do so in a systematic way that could distinguish it from stigma towards HIV infection alone. A way to disentangle the components that make up intersectional stigma will be necessary in order to address its myriad manifestations.

Nearly 40 years into the epidemic and with wider availability of treatment and prevention tools, the persistence of HIV stigma in healthcare settings remains a matter of great urgency. Moreover, combating healthcare stigma in LMICs will require particular attention to the unique combination of resource constraints, high HIV burden and diverse cultural contexts that shape stigma [102, 103, 104, 105, 106, 107, 108]. More recent proposals by Hatzenbuehler *et al*. [109] to regard stigma as a driver of health inequity may inform a social determinants framework to mobilize more novel and interdisciplinary approaches to stigma reduction. By focusing on research conducted in LMIC, this review highlights research crucial to informing impactful and sustainable programmes to tackle the underlying drivers of stigma.

## COMPETING INTERESTS

The authors declare that they have no competing interests.

## AUTHORS’ CONTRIBUTIONS

M.K.S. and S.Y.S. developed the initial concept for the manuscript. M.K.S., R.H.X and S.L.H conducted the literature review, article abstraction and drafted an initial draft. The remaining authors (C.W., W.T., J.D.T., C.W., L.Y., B.Y., H.X., D.L., L.L., B.L.J. and S.Y.S.) edited and contributed content to the final draft. All authors have read and approved the final manuscript.

## Supporting information


**Appendix S1.** Embase search strategy.

## References

[1] Cohen MS , Chen YQ , McCauley M , Gamble T , Hosseinipour MC , Kumarasamy N , et al. Prevention of HIV‐1 Infection with Early Antiretroviral Therapy. N Engl J Med. 2011;365(6):493–505. 21767103 10.1056/NEJMoa1105243 PMC3200068

[2] Rodger AJ , Cambiano V , Bruun T , Vernazza P , Collins S , Degen O , et al. Risk of HIV transmission through condomless sex in serodifferent gay couples with the HIV‐positive partner taking suppressive antiretroviral therapy (PARTNER): final results of a multicentre, prospective, observational study. Lancet (London, England) [Internet]. 2019 Jun 15 [cited 2019 Sep 2];393(10189):2428–38. Available from: http://www.ncbi.nlm.nih.gov/pubmed/31056293 10.1016/S0140-6736(19)30418-0 PMC6584382 31056293

[3] Grant RM , Lama JR , Anderson PL , McMahan V , Liu A , Vargas L , et al. Preexposure Chemoprophylaxis for HIV Prevention in Men Who Have Sex with Men. N Engl J Med. 2010;363(27):2587–99. 21091279 10.1056/NEJMoa1011205 PMC3079639

[4] Troya J , Bascuñana J . Safety and tolerability: current challenges to antiretroviral therapy for the long‐term management of HIV infection. AIDS Rev [Internet]. [cited 2019 Sep 2];18(3):127–37. Available from: http://www.ncbi.nlm.nih.gov/pubmed/27651173 27651173

[5] Goffman E . Stigma: notes on the management of spoiled identity. Englewood Cliffs, NJ: Prentice Hall; 1963 p. 1–147.

[6] Nyblade LC . Measuring HIV stigma: existing knowledge and gaps. Psychol Health Med. 2006;11(3):335–45. 17130069 10.1080/13548500600595178

[7] Golub SA , Gamarel KE . The impact of anticipated HIV stigma on delays in HIV testing behaviors: findings from a community‐based sample of men who have sex with men and transgender women in New York City. AIDS Patient Care STDS [Internet]. 2013 Nov 5 [cited 2018 Oct 30];27(11):621–7. Available from: http://www.liebertpub.com/doi/10.1089/apc.2013.0245 24138486 10.1089/apc.2013.0245 PMC3820140

[8] Wei C , Cheung DH , Yan H , Li J , Shi LE , Raymond HF . The impact of homophobia and HIV stigma on HIV testing uptake among Chinese men who have sex with men: a mediation analysis. J Acquir Immune Defic Syndr. 2016;71(1):87–93. 26334742 10.1097/QAI.0000000000000815 PMC4713338

[9] Santos GM , Beck J , Wilson PA , Hebert P , Makofane K , Pyun T , et al. Homophobia as a barrier to HIV prevention service access for young men who have sex with men. J Acquir Immune Defic Syndr. 2013;63(5):167–70. 10.1097/QAI.0b013e318294de80 24135782

[10] Whetten K , Reif S , Whetten R , Trauma M‐MLK . Mental health, distrust, and stigma among HIV‐positive persons: implications for effective care. Psychosom Med [Internet]. 2008 Jun [cited 2018 Sep 23];70(5):531–8. Available from: https://insights.ovid.com/crossref?an=00006842‐200806000‐00003 18541904 10.1097/PSY.0b013e31817749dc

[11] Logie C , Gadalla TMM . Meta‐analysis of health and demographic correlates of stigma towards people living with HIV. AIDS Care [Internet]. 2009 [cited 2018 Sep 6];21(6):742–53. Available from: https://www.tandfonline.com/doi/full/10.1080/09540120802511877 19806490 10.1080/09540120802511877

[12] Sayles JN , Wong MD , Kinsler JJ , Martins D , Cunningham WE . The association of stigma with self‐reported access to medical care and antiretroviral therapy adherence in persons living with HIV/AIDS. J Gen Intern Med [Internet]. 2009 Oct 4 [cited 2018 Sep 23];24(10):1101–8. Available from: http://link.springer.com/10.1007/s11606‐009‐1068‐8 19653047 10.1007/s11606-009-1068-8 PMC2762503

[13] Zhou YR . Help‐seeking in a context of AIDS stigma: understanding the healthcare needs of people with HIV/AIDS in China. Health Soc Care Community [Internet]. 2009 Mar 1 [cited 2018 Sep 23];17(2):202–8. Available from: http://doi.wiley.com/10.1111/j.1365‐2524.2008.00820.x 19040695 10.1111/j.1365-2524.2008.00820.x

[14] Churcher S . Stigma related to HIV and AIDS as a barrier to accessing health care in Thailand: a review of recent literature. WHO South‐East Asia J public Health [Internet]. 2013 [cited 2018 Sep 23];2(1):12–22. Available from: http://www.ncbi.nlm.nih.gov/pubmed/28612818 28612818 10.4103/2224-3151.115829

[15] Langebeek N , Gisolf EH , Reiss P , Vervoort SC , Hafsteinsdóttir TB , Richter C , et al. Predictors and correlates of adherence to combination antiretroviral therapy (ART) for chronic HIV infection: a meta‐analysis. BMC Med [Internet]. 2014 Aug 21 [cited 2018 Sep 23];12(1):142 Available from: http://www.ncbi.nlm.nih.gov/pubmed/25145556 25145556 10.1186/s12916-014-0142-1 PMC4148019

[16] Li X , Huang L , Wang H , Fennie KP , He G , Williams AB . Stigma mediates the relationship between self‐efficacy, medication adherence, and quality of life among people living with HIV/AIDS in China. AIDS Patient Care STDS [Internet]. 2011 Nov [cited 2018 Sep 23];25(11):665–71. Available from: http://www.ncbi.nlm.nih.gov/pubmed/22023316 22023316 10.1089/apc.2011.0174 PMC3279711

[17] Yang Y , Zhang K‐L , Chan KY , Reidpath DD . Institutional and structural forms of HIV‐related discrimination in health care: A study set in Beijing. AIDS Care [Internet]. 2005 Jul 18 [cited 2020 Feb 26];17 sup2:129–40. Available from: https://www.tandfonline.com/doi/full/10.1080/09540120500119874 10.1080/09540120500119874 16174624

[18] Peltzer K , Pengpid S . Prevalence and associated factors of enacted, internalized and anticipated stigma among people living with HIV in South Africa: Results of the first national survey. HIV/AIDS. 2019;11:275–85. 31807083 10.2147/HIV.S229285 PMC6844236

[19] Lohiniva AL , Kamal W , Benkirane M , Numair T , Abdelrahman M , Saleh H , et al. HIV stigma toward people living with HIV and health providers associated with their care: qualitative interviews with community members in Egypt. J Assoc Nurses AIDS Care. 2016;27(2):188–98. 26718817 10.1016/j.jana.2015.11.007

[20] United Nations . Transforming our World: The 2030 agenda for sustainable development. 2015 [cited 2019 Jun 23]. Available from: https://sustainabledevelopment.un.org/post2015/transformingourworld/publication

[21] Lee RS , Kochman A , Sikkema KJ . Internalized stigma among people living with HIV‐AIDS. AIDS Behav [Internet]. 2002 [cited 2018 Nov 9];6(4):309–19. Available from: http://link.springer.com/10.1023/A:1021144511957

[22] Earnshaw VA , Chaudoir SR . From conceptualizing to measuring HIV stigma: a review of HIV stigma mechanism measures. AIDS Behav. 2009;13(6):1160–77. 19636699 10.1007/s10461-009-9593-3 PMC4511707

[23] Chi P , Li X , Zhao J , Zhao G . Vicious circle of perceived stigma, enacted stigma and depressive symptoms among children affected by HIV/AIDS in China. AIDS Behav. 2014;18(6):1054–62. 24158487 10.1007/s10461-013-0649-z PMC4000575

[24] Link BG , Phelan JC . Stigma and its public health implications. Lancet. 2006;367(9509):528–9. 16473129 10.1016/S0140-6736(06)68184-1

[25] Sengupta S , Banks B , Jonas D , Miles MS , Smith GC . HIV interventions to reduce HIV/AIDS stigma: a systematic review. AIDS Behav. 2011;15(6):1075–87. 21088989 10.1007/s10461-010-9847-0 PMC3128169

[26] Brown L , Macintyre K , Trujillo L . Interventions to reduce HIV/AIDS stigma: what have we learned? AIDS Educ Prev. 2003;15(1):49–69. 12627743 10.1521/aeap.15.1.49.23844

[27] Mak WWS , Mo PKH , Ma GYK , Lam MYY . Meta‐analysis and systematic review of studies on the effectiveness of HIV stigma reduction programs. Soc Sci Med [Internet]. 2017;188:30–40. 28704645 10.1016/j.socscimed.2017.06.045

[28] Stangl AL , Lloyd JK , Brady LM , Holland CE , Baral SD . A systematic review of interventions to reduce HIV‐related stigma and discrimination from 2002 to 2013: how far have we come? J Int AIDS Soc. 2013;16:18734. 24242268 10.7448/IAS.16.3.18734 PMC3833106

[29] Munn Z , Peters MDJ , Stern C , Tufanaru C , McArthur A , Aromataris E . Systematic review or scoping review? Guidance for authors when choosing between a systematic or scoping review approach. BMC Med Res Methodol [Internet]. 2018 Nov 19 [cited 2020 Feb 25];18(1):143 Available from: https://bmcmedresmethodol.biomedcentral.com/articles/10.1186/s12874‐018‐0611‐x 30453902 10.1186/s12874-018-0611-x PMC6245623

[30] Tricco AC , Lillie E , Zarin W , O'Brien KK , Colquhoun H , Levac D , et al. PRISMA extension for scoping reviews (PRISMA‐ScR): checklist and explanation. Ann Intern Med. 2018;169(7):467–73. 30178033 10.7326/M18-0850

[31] Venkat Narayan KM , Miotti PG , Anand NP , Kline LM , Harmston C , Gulakowski R , et al. HIV and noncommunicable disease comorbidities in the era of antiretroviral therapy: a vital agenda for research in low‐and middle‐income country settings. J Acquir Immune Defic Syndr. 2014;67:S2–7. 25117958 10.1097/QAI.0000000000000267

[32] Shao Y , Williamson C . The HIV‐1 epidemic: Low‐ to middle‐income countries. Cold Spring Harb Perspect Med. 2012;2:a007187. 22393534 10.1101/cshperspect.a007187 PMC3282497

[33] Bos AER , Schaalma HP , Pryor JB . Reducing AIDS‐related stigma in developing countries: the importance of theory‐ and evidence‐based interventions. Psychol Heal Med. 2008;13(4):450–60. 10.1080/13548500701687171 18825583

[34] Genberg BL , Kawichai S , Chingono A , Sendah M , Chariyalertsak S , Konda KA , et al. Assessing HIV/AIDS stigma and discrimination in developing countries. AIDS Behav. 2008;12(5):772–80. 18080830 10.1007/s10461-007-9340-6 PMC2745205

[35] Arreola S , Jack GS , Makofane K , Do TD , Ayala G , Altman D , et al. Homophobia as a barrier to HIV prevention service access for young men who have sex with men. Lancet [Internet]. 2013;57(9839):24–34. 10.1097/QAI.0b013e318294de80 24135782

[36] Shannon K , Strathdee SA , Goldenberg SM , Duff P , Mwangi P , Rusakova M , et al. Global epidemiology of HIV among female sex workers: Influence of structural determinants [Internet]. Lancet. 2015 [cited 2018 Jul 9];385(9962):55–71. Available from: http://linkinghub.elsevier.com/retrieve/pii/S0140673614609314 25059947 10.1016/S0140-6736(14)60931-4 PMC4297548

[37] Strathdee SA , Stockman JK . Epidemiology of HIV among injecting and non‐injecting drug users: current trends and implications for interventions. Curr HIV/AIDS Rep. 2010;7(2):99–106. 20425564 10.1007/s11904-010-0043-7 PMC2856849

[38] World bank country and lending groups [Internet]. 2019 [cited 2018 Jul 9]. Available from: https://datahelpdesk.worldbank.org/knowledgebase/articles/906519‐world‐bank‐country‐and‐lending‐groups

[39] Developing/low‐middle income countries searches [Internet]. Chapel Hill, NC; 2016 [cited 2018 Jul 9]. Available from: https://guides.lib.unc.edu/c.php?g=8369&p=784120

[40] Scherer RW , Saldanha IJ . How should systematic reviewers handle conference abstracts? A view from the trenches. Syst Rev. 2019;8(1):4–9. 31699124 10.1186/s13643-019-1188-0 PMC6836535

[41] Ouzzani M , Hammady H , Fedorowicz Z , Elmagarmid A . Rayyan‐a web and mobile app for systematic reviews. Syst Rev [Internet]. 2016;5(1):1–10. 27919275 10.1186/s13643-016-0384-4 PMC5139140

[42] Nyblade L , Stangl A , Weiss E , Ashburn K . Combating HIV stigma in health care settings: what works? J Int. AIDS Soc. 2009;12:15. 10.1186/1758-2652-12-15 PMC2731724 19660113

[43] Tufanaru C , Munn Z , Aromataris E , Campbell J , Hopp L . Chapter 3: Systematic reviews of effectiveness In: Aromataris E, Munn Z editors. Joanna briggs institute reviewer’s manual [Internet]. North Adelaide; 2017 [cited 2018 Jul 9]. Available from: https://joannabriggs.org/critical_appraisal_tools

[44] Geibel S , Hossain SMI , Pulerwitz J , Sultana N , Hossain T , Roy S , et al. Stigma reduction training improves healthcare provider attitudes toward, and experiences of, young marginalized people in Bangladesh. J Adolesc Heal. 2017;60:S35–44. 10.1016/j.jadohealth.2016.09.026 28109339

[45] Diesel H , Ercole P , Taliaferro D . Knowledge and perceptions of HIV/AIDS among cameroonian nursing students. Int J Nurs Educ Scholarsh. 2013;10(1):209–18. 10.1515/ijnes-2012-0036 24047598

[46] Mbeba MM , Kaponda CP , Jere DL , Kachingwe SI , Crittenden KS , McCreary LL , et al. Peer group intervention reduces personal HIV risk for Malawian health workers. J Nurs Sch. 2011;43(1):72–81. 10.1111/j.1547-5069.2011.01384.x PMC3073810 21342427

[47] Uys L , Chirwa M , Kohi T , Greeff M , Naidoo J , Makoae L , et al. Evaluation of a health setting‐based stigma intervention in five African countries. AIDS Patient Care STDS. 2009;23(12):1059–66. 20025515 10.1089/apc.2009.0085 PMC2832642

[48] Ezedinachi E , Ross M , Meremiku M , Essien E , Edem C , Ekure E , et al. The impact of an intervention to change health workers’ HIV/AIDS attitudes and knowledge in Nigeria: a controlled trial. Public Health. 2002;116(2):106–12. 11961679 10.1038/sj.ph.1900834

[49] Pulerwitz J , Oanh KTH , Akinwolemiwa D , Ashburn K , Nyblade L . Improving hospital‐based quality of care by reducing HIV‐related stigma: evaluation results from Vietnam. AIDS Behav. 2015;19(2):246–56. 25382350 10.1007/s10461-014-0935-4

[50] Li L , Lin C , Guan J . Using standardized patients to evaluate hospital‐based intervention outcomes. Int J Epidemiol. 2014;43(3):897–903. 24369433 10.1093/ije/dyt249 PMC4052130

[51] Wang D , Operario D , Hong Q , Zhang H , Coates TJ . Intervention to train physicians in rural China on HIV/STI knowledge and risk reduction counseling: Preliminary findings. AIDS Care. 2009;21(4):468–72. 19266406 10.1080/09540120802290357 PMC2853945

[52] Williams AB , Wang H , Burgess J , Wu C , Gong Y , Li Y . Effectiveness of an HIV/AIDS educational programme for Chinese nurses. J Adv Nurs. 2006;53(6):710–20. 16553679 10.1111/j.1365-2648.2006.03777.x

[53] Lohiniva AL , Benkirane M , Numair T , Mahdy A , Saleh H , Zahran A , et al. HIV stigma intervention in a low‐HIV prevalence setting: a pilot study in an Egyptian healthcare facility. AIDS Care. 2016;28(5):644–52. 26717980 10.1080/09540121.2015.1124974

[54] Pisal H , Sutar S , Sastry J , Kapadia‐Kundu N , Joshi A , Joshi M , et al. Nurses’ health education program in India increases HIV knowledge and reduces fear. J Assoc Nurses AIDS Care. 2007;18(6):32–43. 10.1016/j.jana.2007.06.002 17991597

[55] Shah SM , Heylen E , Srinivasan K , Perumpil S , Ekstrand ML . Reducing HIV stigma among nursing students: a brief intervention. West J Nurs Res. 2014;36(10):1323–37. 24569699 10.1177/0193945914523685 PMC4459739

[56] Edwards N , Kaseje D , Kahwa E , Klopper HC , Mill J , Webber J , et al. The impact of leadership hubs on the uptake of evidence‐informed nursing practices and workplace policies for HIV care: A quasi‐experimental study in Jamaica, Kenya, Uganda and South Africa. Implement Sci [Internet]. 2016;11(1):110. 27488735 10.1186/s13012-016-0478-3 PMC4973110

[57] Kaponda CP , Jere DL , Chimango JL , Chimwaza AF , Crittenden KS , Kachingwe SI , et al. Impacts of a peer‐group intervention on HIV‐related knowledge, attitudes, and personal behaviors for urban hospital workers in Malawi Chrissie. J Assoc Nurses AIDS Care. 2009;20(3):230–42. 19427600 10.1016/j.jana.2008.12.005 PMC4177099

[58] Nanayakkara GN , Choi E‐O . Effectiveness of AIDS education program on nursing students’ AIDS knowledge and AIDS attitudes in Sri Lanka. J Nurs Educ Pract [Internet]. 2016;8(6):1.

[59] Nwuba C . Structural interventions to reduce stigmatization and increase uptake of HIV treatment services. Sex Transm Infect. 2013;89:A327.

[60] Allam Rr , Oruganti G , Uthappa Ck , Yeldandi V . Private public partnership for stigma free HIV service delivery in APAIDSCON network in India. Int J Infect Dis. [Internet]. 2016;45:252.

[61] Kiragu K , Nyumbu M , Ngulube TJ , Njobvu P , Mwaba C , Kalimbwe A , et al. Caring for caregivers: an HIV/AIDS workplace intervention for hospital staff in Zambia; Horizons final report. Washington DC: 2008.

[62] Mahendra VS , Gilborn L , George B , Samson L , Mudoi R , Jadav S , et al. Reducing AIDS‐related Stigma and Discrimination in Indian Hospitals. Horizons Final Report. New Delhi; 2006.

[63] Nyblade L , Addo NA , Mingkwan P , Vormawor R , Stewart C , Gyamera E , et al. Understanding and responding to stigma and discrimination in health facilities in Ghana: Intervention endline report [Internet]. Washington DC: 2018 [cited 2019 Sept 22]. Available from: http://www.healthpolicyplus.com/ns/pubs/10267‐10481_TanzaniaStigmat.pdf

[64] Health Policy Project . Measuring HIV stigma and discrimination among health facility staff: standardized brief questionnaire [Internet]. 2013 [cited 2019 Sept 22]. Available from: http://www.healthpolicyproject.com/index.cfm?id=StigmaPackage

[65] Nyblade L , Jain A , Benkirane M , Li L , Lohiniva AL , McLean R , et al. A brief, standardized tool for measuring HIV‐related stigma among health facility staff: results of field testing in China, Dominica, Egypt, Kenya, Puerto Rico and St. Christopher & Nevis. J Int AIDS Soc. 2013;16 3 Suppl 2:1–11. 24242266 10.7448/IAS.16.3.18718 PMC3833189

[66] Stackpool‐Moore L , Singh A . Sexual and reproductive health and rights, and HIV 101 workshop guide: A guide to facilitating a workshop on linking up HIV and sexual and reproductive health and rights with young key populations [Internet]. Brighton, UK; 2015 [cited 2019 Sept 22]. Available from: https://hivhealthclearinghouse.unesco.org/library/documents/sexual‐and‐reproductive‐health‐and‐rights‐and‐hiv‐101‐workshop‐guide‐guide

[67] Dubbert PM , Kemppainen JK , White‐Taylor D . Development of a measure of willingness to provide nursing care to AIDS patients. Nurs Adm Q. 1994;18(2):16–21. 8159326

[68] Froman RD , Owen SV , Daisy C . Development of a measure of attitudes toward persons with AIDS. Image J Nurs Scholarsh [Internet]. 1992 Jun 1 [cited 2019 Jul 31];24(2):149–52. Available from: http://doi.wiley.com/10.1111/j.1547‐5069.1992.tb00240.x 10.1111/j.1547-5069.1992.tb00240.x 1601457

[69] Jemmott JB , Freleicher J , Jemmott LS . Perceived risk of infection and attitudes toward risk groups: Determinants of nurses’ behavioral intentions regarding AIDS patients. Res Nurs Health [Internet]. 1992 Aug 1 [cited 2019 Jul 31];15(4):295–301. 1496153 10.1002/nur.4770150408

[70] United States Agency for International Development , Inter‐Agency Working Group on Stigma and Discrimination . HIV‐related stigma and discrimination indicators development workshop report. 2004.

[71] Li L , Wu Z , Wu S , Zhao Y , Jia M , Yan Z. HIV‐related stigma in health care settings: a survey of service providers in China. AIDS Patient Care STDS [Internet]. 2007 Oct [cited 2014 Aug 11];21(10):753–62. Available from: http://www.pubmedcentral.nih.gov/articlerender.fcgi?artid=2795451&tool=pmcentrez&rendertype=abstract 17949274 10.1089/apc.2006.0219 PMC2795451

[72] Rogers EM . Diffusion of innovations. 4th ed. Everett M. Rogers; 1995.

[73] Froman RD , Owen SV . Measuring attitudes toward persons with AIDS: the AAS‐G as an alternate form of the AAS. Sch Inq Nurs Pract. 2001;15(2):161‐74. 11695492

[74] Jemmott LS , Jemmott JB , Cruz‐Couins M . Predicting AIDS patient care intentions among nursing students. Nurs Res. 1992;41(3):172–7. 1584661

[75] Wu S , Li L , Wu Z , Liang L‐J , Cao H , Yan Z , et al. A brief HIV stigma reduction intervention for service providers in China. AIDS Patient Care STDS [Internet]. 2008 [cited 2020 Mar 5];22(6):513–20. Available from http://www.liebertonline.com/doi/abs/10.1089/apc.2007.0198 18462076 10.1089/apc.2007.0198 PMC2700336

[76] Oanh KTH , Ashburn K , Pulerwitz J , Ogden J , Nyblade L . Improving hospital‐based quality of care in Vietnam by reducing HIV related stigma and discrimination: a horizons final report. Washington DC; 2008.

[77] Mahendra VS , Gilborn L , Bharat S , Mudoi RJ , Gupta I , George B , et al. Understanding and measuring AIDS‐related stigma in health care settings: a developing country perspective. Sahara J [Internet]. 2007 Aug [cited 2019 Jul 31];4(2):616–25. Available from: http://www.tandfonline.com/doi/abs/10.1080/17290376.2007.9724883 18071613 10.1080/17290376.2007.9724883

[78] Herek GM , Capitanio JP . AIDS stigma and sexual prejudice. Am Behav Scientist. 1999;42(7):1130–47.

[79] Steward WT , Herek GM , Ramakrishna J , Bharat S , Chandy S , Wrubel J , et al. HIV‐related stigma: adapting a theoretical framework for use in India. Soc Sci Med. 2008;67(8):1225–35. 18599171 10.1016/j.socscimed.2008.05.032 PMC2603621

[80] Ekstrand ML , Ramakrishna J , Bharat S , Heylen E . Prevalence and drivers of HIV stigma among health providers in urban India: implications for interventions. J Int AIDS Soc [Internet]. 2013 Nov 13 [cited 2018 Dec 23];16:18717 Available from: http://www.ncbi.nlm.nih.gov/pubmed/24242265 24242265 10.7448/IAS.16.3.18717 PMC3833193

[81] Uys LR , Holzemer WL , Chirwa ML , Dlamini PS , Greeff M , Kohi TW , et al. The development and validation of the HIV/AIDS Stigma Instrument – Nurse (HASI‐N). AIDS Care [Internet]. 2009 Feb 19 [cited 2019 Jul 31];21(2):150–9. Available from: https://www.tandfonline.com/doi/full/10.1080/09540120801982889 19229683 10.1080/09540120801982889 PMC2716132

[82] Talashek ML , Kaponda CPN , Jere DL , Kafulafula U , Mbeba MM , McCreary LL , et al. Identifying what rural health workers in Malawi need to become HIV prevention leaders. J Assoc Nurses AIDS Care. 2007;18(4):41–50. 17662923 10.1016/j.jana.2007.05.007

[83] Kachingwe SI , Norr K , Kaponda CPN , Norr J , Mbweza E , Magai D . Preparing teachers as HIV/AIDS prevention leaders in Malawi: evidence from focus groups. Int Electron J Health Educ [Internet]. 2005 [cited 2020 Mar 2];8:193–204. Available from: http://search.ebscohost.com/login.aspx?direct=true&AuthType=cookie,ipshib&db=rzh&AN=2009073731&site=ehost‐live

[84] Norr K , Tlou S , Moeti M . Impact of peer group education on HIV prevention among women in Botswana. Health Care Women Int [Internet]. 2004 Mar [cited 2019 Sep 9];25(3):210–26. Available from: http://www.tandfonline.com/doi/abs/10.1080/07399330490272723 15195767 10.1080/07399330490272723

[85] McElmurry B , Keeney G . Primary health care. Annu Rev Nurs Res. 1999;17:241–68. 10418660

[86] Holzemer WL , Uys LR , Chirwa ML , Greeff M , Makoae LN , Kohi TW , et al. Validation of the HIV/AIDS Stigma Instrument—PLWA (HASI‐P). AIDS Care [Internet]. 2007 Sep 24 [cited 2019 Aug 2]19(8):1002–12. Available from: https://www.tandfonline.com/doi/full/10.1080/09540120701245999 17851997 10.1080/09540120701245999

[87] Tanzania Stigma‐Indicators Field Test Group . Working report: Measuring HIV stigma: results of a field test in Tanzania. Washington DC 2005.

[88] Kidd R , Clay S . Understanding and challenging HIV stigma: Toolkit for action [Internet]. Brighton, UK; 2003 [cited 2020 Mar 2]. Available from: https://www.icrw.org/wp‐content/uploads/2016/10/Understanding‐and‐Challenging‐HIV‐Stigma‐Toolkit‐for‐Action.pdf

[89] The World Bank . The world by region [Internet]. 2018 [cited 2020 Mar 2]. Available from: http://datatopics.worldbank.org/sdgatlas/the‐world‐by‐region.html

[90] Li L , Wu Z , Liang L‐J , Lin C , Guan J , Jia M , et al. Reducing HIV‐related stigma in health care settings: a randomized controlled trial in China. Am J Public Health [Internet]. 2013 [cited 2020 Mar 5];103(2):286–92. Available from http://www.pubmedcentral.nih.gov/articlerender.fcgi?artid=3556241&tool=pmcentrez&rendertype=abstract 23237175 10.2105/AJPH.2012.300854 PMC3556241

[91] Kharsany ABM , Karim QA . HIV infection and AIDS in Sub‐Saharan Africa: current status, challenges and opportunities. Open AIDS J [Internet]. 2016 [cited 2019 Sep 5];10:34–48. Available from: http://www.ncbi.nlm.nih.gov/pubmed/27347270 27347270 10.2174/1874613601610010034 PMC4893541

[92] Global AIDS Update . Miles to go: the response to HIV in Eastern Europe and Central Asia [Internet]. Geneva, Switzerland; 2018 [cited 2020 Mar 2]. Available from: https://www.unaids.org/sites/default/files/media_asset/miles‐to‐go_eastern‐europe‐and‐central‐asia_en.pdf

[93] Cohen J . Russia’s HIV/AIDS epidemic is getting worse, not better. Science [Internet]. 2018 Jun 11 [cited 2019 Sep 6]; Available from: http://www.sciencemag.org/news/2018/06/russia‐s‐hivaids‐epidemic‐getting‐worse‐not‐better

[94] García PJ , Bayer A , Cárcamo CP . The Changing face of HIV in Latin America and the Caribbean. Curr HIV/AIDS Rep [Internet]. 2014 Jun 15 [cited 2019 Sep 5];11(2):146–57. Available from: http://link.springer.com/10.1007/s11904‐014‐0204‐1 24824881 10.1007/s11904-014-0204-1 PMC4136548

[95] De Boni R Veloso VG , Grinsztejn B . Epidemiology of HIV in Latin America and the Caribbean. Curr Opin HIV AIDS [Internet]. 2014 Mar [cited 2019 Sep 5];9(2):192–8. Available from: http://content.wkhealth.com/linkback/openurl?sid=WKPTLP:landingpage&an=01222929‐201403000‐00013 24356327 10.1097/COH.0000000000000031

[96] Nyblade LC , Addo N , K A , Gyamera E , C S , Jachinthe S , et al. Reducing health worker stigma and discrimination is critical to reaching 90‐90‐90 targets and is possible: evaluation results of a whole‐facility approach in Ghana. In: 22nd International AIDS Conference, Amsterdam, Netherlands. 2018 Jul 23‐27. p. 171.

[97] Nyblade L , MacQuarrie K . Can We Measure HIV/AIDS‐Related Stigma and Discrimination? Current Knowledge about Quantifying Stigma in Developing Countries. United States Agency for International Development. 2006 [cited 2019 Jun 23]. Available from: https://www.icrw.org/wp‐content/uploads/2016/10/Can‐We‐Measure‐HIV‐Stigma‐and‐Discrimination.pdf

[98] Harkness JA, Braun M, Edwards B, Johnson TP, Lyberg L, Mohler PP, et al. editors, Survey methods in multicultural, multinational, and multiregional contexts. Vol. 552 Hoboken (NJ): Wiley; 2010.

[99] Earnshaw VA , Smith LR , Chaudoir SR , Amico KR , Copenhaver MM . HIV stigma mechanisms and well‐being among PLWH: A test of the HIV Stigma Framework. AIDS Behav. 2013;17(5):1785–95. 23456594 10.1007/s10461-013-0437-9 PMC3664141

[100] Homer CSE . Using the Zelen design in randomized controlled trials: debates and controversies. J Adv Nurs. 2002;38(2):200–7. 11940133 10.1046/j.1365-2648.2002.02164.x

[101] Schellings R , Kessels AG , ter Riet G , Knottnerus JA , Sturmans F . Randomized consent designs in randomized controlled trials: systematic literature search. Contemp Clin Trials. 2006;27(4):320–32. 16388991 10.1016/j.cct.2005.11.009

[102] Latypov A , Rhodes T , Reynolds L . Prohibition, stigma and violence against men who have sex with men: effects on HIV in Central Asia. Centr Asian Surv [Internet]. 2013 Mar [cited 2019 Sep 6];32(1):52–65. Available from: http://www.tandfonline.com/doi/abs/10.1080/02634937.2013.768059

[103] Smolak A , El‐Bassel N . Multilevel stigma as a barrier to HIV testing in central asia: a context quantified. AIDS Behav [Internet]. 2013 Oct 1 [cited 2019 Sep 6];17(8):2742–55. Available from: http://link.springer.com/10.1007/s10461‐013‐0571‐4 23904147 10.1007/s10461-013-0571-4

[104] Rechel B . HIV/AIDS in the countries of the former Soviet Union: societal and attitudinal challenges. Cent Eur J Public Health [Internet]. 2010;18(2):110–5. 20939262 10.21101/cejph.a3583

[105] Calabrese SK , Burke SE , Dovidio JF , Levina OS , Uusküla A , Niccolai LM , et al. Internalized HIV and drug stigmas: interacting forces threatening health status and health service utilization among people with HIV who inject drugs in St. Petersburg, Russia. AIDS Behav [Internet]. 2016 Jan 7 [cited 2019 Sep 6];20(1):85–97. Available from: http://link.springer.com/10.1007/s10461‐015‐1100‐4 26050155 10.1007/s10461-015-1100-4 PMC4793904

[106] Burke SE , Calabrese SK , Dovidio JF , Levina OS , Uusküla A , Niccolai LM , et al. A tale of two cities: Stigma and health outcomes among people with HIV who inject drugs in St. Petersburg, Russia and Kohtla‐Järve, Estonia. Soc Sci Med [Internet]. 2015 Apr 1 [cited 2019 Sep 6];130:154–61. Available from: https://www.sciencedirect.com/science/article/pii/S027795361500101X 25703668 10.1016/j.socscimed.2015.02.018 PMC4363273

[107] Rogers SJ , Tureski K , Cushnie A , Brown A , Bailey A , Palmer Q . Layered stigma among health‐care and social service providers toward key affected populations in Jamaica and The Bahamas. AIDS Care [Internet]. 2014 May 4 [cited 2019 Sep 6];26(5):538–46. Available from: http://www.tandfonline.com/doi/abs/10.1080/09540121.2013.844762 24125067 10.1080/09540121.2013.844762

[108] Surkan PJ , Mukherjee JS , Williams DR , Eustache E , Louis E , Jean‐Paul T , et al. Perceived discrimination and stigma toward children affected by HIV/AIDS and their HIV‐positive caregivers in central Haiti. AIDS Care [Internet]. 2010 Jul 15 [cited 2019 Sep 6];22(7):803–15. Available from: https://www.tandfonline.com/doi/full/10.1080/09540120903443392 20635244 10.1080/09540120903443392 PMC3017757

[109] Hatzenbuehler ML , Phelan JC , Link BG . Stigma as a fundamental cause of population health inequalities. Am J Public Health. 2013;103(5):813–21. 23488505 10.2105/AJPH.2012.301069 PMC3682466

