# Supplementary material for: Combating HIV stigma in low‐ and middle‐income healthcare settings: a scoping review
Source: J Int AIDS Soc. 2020 Aug 26;23(8):e25553. doi: 10.1002/jia2.25553 (PMC7448195; doi:10.1002/jia2.25553)
Supplement: Supplementary file 1 — Appendix S1. Embase search strategy. [file JIA2-23-e25553-s001.docx]

**Appendix 1. Embase Search Strategy**

| **Concept [AND]** | **Emtree Terms & Keywords (ti, ab, de fields) [OR]** |
| --- | --- |
| HIV/AIDS | HIV  HIV/AIDS  human immunodeficiency virus*  AIDS  acquired immunodeficiency syndrome  seropositive*  serostatus  PLWH  PLWA  PLWHA  HIV infection |
| Low and Middle Income Countries | Afghanistan  Albania  Algeria  American Samoa  Angola or Armenia  Azerbaijan  Bangladesh  Belarus  Byelarus  Belorussia  Belize  Benin  Bhutan  Bolivia  Bosnia  Botswana  Brazil  Bulgaria  Burma  Burkina Faso  Burundi  Cabo Verde  Cape verde  Cambodia  Cameroon  Central African Republic  Chad  China  Colombia  Comoros  Comores  Comoro  Congo  Costa Rica  Cote d Ivoire  Cuba  Djibouti  Dominica  Dominican Republic  Ecuador  Egypt  El Salvador  Eritrea  Ethiopia  Fiji  Gabon  Gambia  Gaza  Georgia Republic  Georgian  Ghana  Grenada  Grenadines  Guatemala  Guinea  Guinea Bisau  Guyana  Haiti  Herzegovina  Hercegovina  Honduras  India  Indonesia  Iran  Iraq  Jamaica  Jordan  Kazakhstan  Kenya  Kiribati  Korea  Kosovo  Kyrgyz  Kirghizia  Kirghiz  Kirgizstan  Kyrgyzstan  Lao PDR  Laos  Lebanon  Lesotho  Liberia  Libya  Macedonia  Madagascar  Malawi  Malay  Malaya  Malaysia  Maldives  Mali  Marshall Islands  Mauritania  Mauritius  Mexico  Micronesia  Moldova  Mongolia  Montenegro  Morocco  Mozambique  Myanmar  Namibia  Nauru  Nepal  Nicaragua  Niger  Nigeria  Pakistan  Papua New Guinea  Paraguay  Peru  Philippines  Phillippines  Philipines  Phillipines  Principe  Romania  Russia*  Rwanda  Ruanda  Samoa  Sao Tome  Senegal  Serbia  Sierra Leone  Solomon Islands  Somalia  South Africa  South Sudan  Sri Lanka  St Lucia  St Vincent  Sudan  Surinam  Suriname  Swaziland  Syria  Syrian Arab Republic  Tajikistan  Tadzhikistan  Tadjikistan  Tadzhik  Tanzania  Thailand  Timor  Togo  Tonga  Tunisia  Turkey  Turkmen  Turkmenistan  Tuvalu  Uganda  Ukraine  Uzbek  Uzbekistan  Vanuatu  Venezuela  Vietnam  West Bank  Yemen  Zambia  Zimbabwe  deprived countr*  deprived nation*  deprived world*  developing countr*  developing econom*  developing nation*  developing world*  LAMI countr*  less developed countr*  less developed econom*  less developed nation*  less developed world*  lesser developed countr*  lesser developed econom*  lesser developed nation*  lesser developed world*  LMIC*  low gdp  low gnp  low gross domestic  low gross national  low income countr*  low income econom*  low income nation*  lower gdp  lower gnp  lower gross domestic  lower gross national  lower income countr*  lower income econom*  lower income nation*  middle income countr*  middle income econom*  middle income nation*  poor countr*  poor econom*  poor nation*  poor world*  poorer countr*  poorer econom*  poorer nation*  poorer world*  third world  transitional countr*  transitional econom*  under developed countr*  under developed econom*  under developed nation*  under developed world*  under served countr*  under served nation*  under served world*  underdeveloped countr*  underdeveloped econom*  underdeveloped nation*  underdeveloped world*  underserved countr*  underserved nation*  underserved world*  least developed countr*  least developed nation*  underprivileged countr*  underprivileged nation*  disadvantaged countr*  disadvantaged nation*  global south  low middle income countr*  low middle income nation*  LIC  resource poor countr*  resource poor nation*  emergent countr*  emergent nation*  emerging countr*  emerging nation*  underresourced countr*  underresourced nation*  low SES countr*  low SES nation*  low socioeconomic countr*  low socioeconomic nation*  low socio economic countr*  low socio economic nation*  impoverished countr*  impoverished nation*  resource constrain* countr*  resource constrain* nation* |
| Stigma | stigma*  prejudi*  discriminat*  stereotyp*  marginaliz*  social avoidance  social acceptance |
| Intervention* | intervention*  epidemiol*  clinical trial*  controlled trial*  experiment*  quasi experiment*  single blind*  double blind*  non randomi#ed  nonrandomi#ed  randomi#ed  crossover procedure  RCT  pilot*  prospective  retrospective  time series  pre test  pretest  post test  posttest  cohort  cross sectional  quantitative |

*The search for intervention terms also included the pt field
